# Cost-utility analysis of Palivizumab for Respiratory Syncytial Virus infection prophylaxis in preterm infants: update based on the clinical evidence in Spain

**DOI:** 10.1186/s12879-017-2803-0

**Published:** 2017-10-17

**Authors:** M. Sanchez-Luna, R. Burgos-Pol, I. Oyagüez, J. Figueras-Aloy, M. Sánchez-Solís, F. Martinón-Torres, X. Carbonell-Estrany

**Affiliations:** 1 Hospital General Universitario Gregorio Marañón, Madrid, Spain; 2 Pharmacoeconomics & Outcomes Research Iberia (PORIB), Paseo Joaquín Rodrigo 4-I, Pozuelo de Alarcón, 28224 Madrid, Spain; 3 Hospital Clinic, Catedratic de Pediatria, Universitat de Barcelona, Barcelona, Spain; 4 Hospital Universitario Virgen de la Arrixaca, Murcia, Spain; 5 Hospital Clínico Universitario de Santiago, Santiago de Compostela, Spain; 6 Hospital Clinic, Institut d’Investigacions Biomediques August Pi Suñer (IDIBAPS), Barcelona, Spain

**Keywords:** Cost-effectiveness, Preterm infants, Palivizumab, RSV infection, Recurrent wheezing

## Abstract

**Background:**

This study aimed at estimating the efficiency of palivizumab in the prevention of Respiratory Syncytial Virus (RSV) infection and its sequelae in preterm infants (32^day 1^-35^day 0^weeks of gestational age –wGA-) in Spain.

**Methods:**

A decision-tree model was developed to compare health benefits (Quality Adjusted Life Years-QALYs) and costs of palivizumab versus a non-prophylaxis strategy over 6 years. A hypothetical cohort of 1,000 preterm infants, 32^day 1^-35^day 0^ wGA (4.356 kg average weight) at the beginning of the prophylaxis (15 mg/kg of palivizumab; 3.88 average number of injections per RSV season) was analysed.

The model considered the most recent evidence from Spanish observational and epidemiological studies on RSV infection: the FLIP II study provided hospital admission and Intensive Care Unit (ICU) admission rates; in-hospital mortality rate was drawn from an epidemiological study from 2004 to 2012; recurrent wheezing rates associated to RSV infection from SPRING study were adjusted by the evidence on the palivizumab effect from clinical trials. Quality of life baseline value, number of hospitalized infants and the presence of recurrent wheezing over time were granted to estimate QALYs.

National Health Service and societal perspective (included also recurrent wheezing indirect cost) were analysed. Total costs (€, 2016) included pharmaceutical and administration costs, hospitalization costs and recurrent wheezing management annual costs. A discount rate of 3.0% was applied annually for both costs and health outcomes.

**Results:**

Over 6 years, the base case analysis showed that palivizumab was associated to an increase of 0.0731 QALYs compared to non-prophylaxis. Total costs were estimated in €2,110.71 (palivizumab) and €671.68 (non-prophylaxis) from the National Health System (NHS) perspective, resulting in an incremental cost utility ratio (ICUR) of €19,697.69/QALYs gained (prophylaxis vs non-prophylaxis). Results derived from the risk-factors population subgroups analysed were in line with the total population results. From the societal perspective, the incremental cost associated to palivizumab decreased to an €1,253.14 (ICUR = €17,153.16€/QALYs gained for palivizumab vs non-prophylaxis). One-way and probabilistic sensitivity analyses confirmed the robustness of the model.

**Conclusions:**

The prophylaxis with palivizumab is efficient for preventing from RSV infections in preterm infants 32^day 1^-35^day 0^ wGA in Spain.

**Electronic supplementary material:**

The online version of this article (10.1186/s12879-017-2803-0) contains supplementary material, which is available to authorized users.

## Background

Respiratory Syncytial Virus (RSV) is the most common cause of acute lower respiratory infections in infants and young children worldwide [1]. Recently, it has been associated to 12–63% of acute respiratory infections in western countries [2].

RSV infections also remains the most important reason of hospital admission among previously healthy infants during the first year of life [3, 4]. In western countries, 70–90% of hospital admissions in acute RSV infection occur in infants aged < 12 months [2]. In fact, it is especially relevant during the first few months of life [5], since they mainly affect to infants ≤ 6 months of age [2]. Moreover, around a fifth (18–22%) of young children RSV-infected are often admitted to Intensive Care Units (ICU) during their hospital stay [6, 7], showing an increasing trend in recent years [6].

In industrialized and developing countries, RSV infections represent the leading cause of death associated with respiratory infections. The estimated global neonatal (0–27 days) and post-neonatal (27–365 days) mortality is around 2–3% and 6–7% respectively which reflects a significant burden of disease in these countries [8].

Some risk factors are related to hospitalizations in RSV-infected children, such as male sex, age < 6 moths, birth during the first half of the RSV season, crowding/siblings and day-care exposure [2]. Prematurity is considered as an independent risk factor of acute respiratory infection [9] and hospital admission among young children (< 5 years old) who experienced respiratory infections [7]. Those premature infants at lower weeks of gestational age (wGA) are at a higher risk of hospitalization [10], including the specific group of pre-term infants 33–36 wGA [11]. Finally, the prevalence of the risk factors as prematurity (< 37 wGA), heart disease and bronchopulmonary dysplasia, is around 20% in children RSV-infected admitted to hospital [11].

Moreover, recurrent wheezing is considered as one of the main long-term RSV infection related outcomes [2]. It has been also associated to the clinical severity of the illness, as children younger than 2 years old are at higher risk of developing recurrent wheezing compared to non-hospitalized young children [12].

In Spain, the burden of disease in RSV-infected pre-term infants 32^day 1^-35^day 0^ has been analysed through different observational studies. The FLIP I [13] and FLIP II [14] studies allowed to identify and validate the risk factors linked to hospital admission in the Spanish population. Later, the SPRING study [15] estimated the long-term effects in terms of recurrence of wheezing on this group of preterm infants; and in addition to that, the in-hospital mortality rates associated to this population group, has been recently published [3].

Palivizumab is a monoclonal antibody used to prevent serious lower respiratory tract disease caused by RSV that would require hospitalization in children who are < 6 months old and were born ≤ 35 wGA [16]. Observational studies conducted in western countries have shown the effect of palivizumab not only on reducing hospitalizations in preterm infants 32^day 1^-35^day 0^ RSV-infected but also on preventing recurrent wheezing during 12–36 months of follow-up [17–19].

The efficiency of palivizumab on preterm infants has been widely examined in Europe [20–24] and United States [25–27] during the last decade. However, important methodological differences among studies (i.e. wGA of preterm infants analysed, time horizon, inclusion of sequelae) might explained the variations of cost-effectiveness ratios obtained. To date, all economic evaluations developed in Spain focused on modelling the effect of palivizumab on avoiding hospitalizations [28–30], but none have included the additional existing evidence on reducing its long-term effects in preterm infants 32^day 1^-35^day 0^ RSV-infected. Besides, due to changes in the current price of palivizumab in the Spanish market and the fact that new country-specific evidence on hospitalizations and mortality rates in the specific group of preterm infants mentioned before is available since the last economic evaluation developed in Spain [28] an update of the efficiency of palivizumab at local level would be required.

Thus, the aim of this study is to assess the efficiency of palivizumab-based prophylaxis strategy in prevention of RSV infection and its sequelae in preterm infants (32^day 1^-35^day 0^ wGA) compared to a strategy of non-prophylaxis, considering the most recent clinical evidence available for Spain.

## Methods

### Model structure

**Fig. 1:**
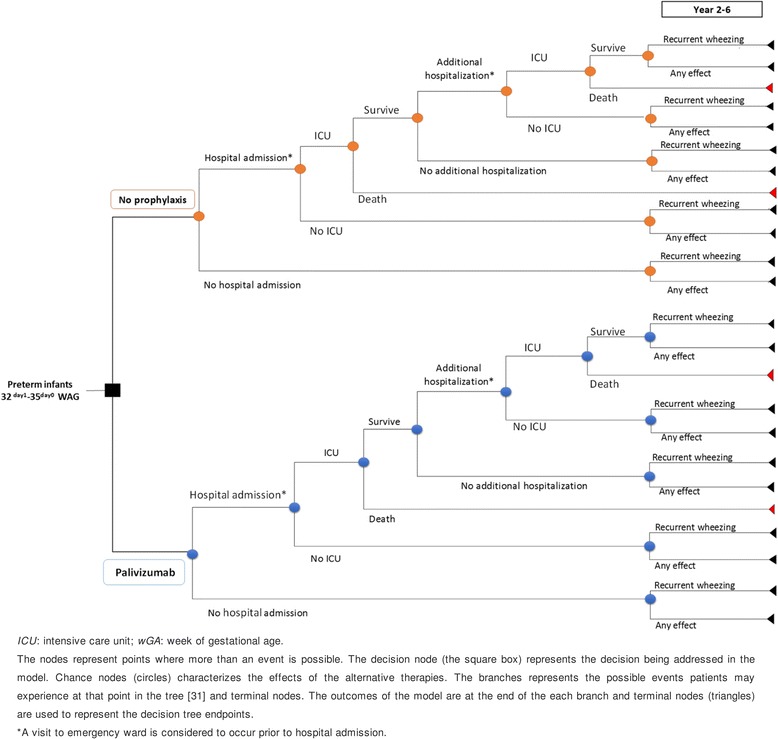
Decision tree model of palivizumab versus non-prophylaxis in the prevention of RSV infection in preterm infants

A decision tree analytic model was developed in Microsoft Excel 2013, to determine health outcomes and costs associated to RSV infections and its sequelae in Spanish preterm infants (32^day 1^ – 35^day 0^ wGA). A prophylaxis strategy for RSV infection consisting of palivizumab administration was compared to a non-prophylaxis strategy. At the end of the path, each branch of the decision tree provided the outcomes of the model (Fig. 1).

The main effectiveness outcome was the quality-adjusted life years (QALYs), which adjusts life years gained (LYG) by the utility value (ranges from 0 to 1) [31]. Costs along with QALYs were used to calculate the incremental cost-utility ratio (ICUR); it is the ratio of the incremental cost of an additional QALY gained when comparing palivizumab vs non-prophylaxis strategy. Model assumptions and parameters of resource use were decided in consultation with an advisory group.

The analysis was carried out from a National Health Service (NHS) (only direct health care costs were considered) and societal perspective (included also indirect costs). The time horizon was fixed in 6 years according to the maximum period of the existing evidence on recurrent wheezing consequences among RSV-infected preterm infants [15]. Costs and outcomes were discounted at 3.0% annually for the base case [32].

### Patient population

The analysis was carried out with a hypothetical cohort of 1,000 preterm infants (32^day 1^ – 35^day 0^ wGA). Further, 3 different population subgroups were defined according to presence of risk factors associated with RSV infection requiring hospitalization: subgroup A (2 major risk factors and 2 minor risk factors); subgroup B (2 major risk factors and 1 minor risk factors); subgroup C (2 major factors). Major factors included chronological age less than 10 weeks at the beginning of RSV season or being born during the first 10 weeks of the season; school-age siblings or day-care attendance whereas minor factors included mother smoking during pregnancy and male gender [33]**.**

### Clinical inputs

Clinical studies in Spanish population [3, 14, 15, 33] were used to determine the model parameters estimates (see Table 1). The probability of hospitalization (1.30% -palivizumab- vs 4.10% -non-prophylaxis-) were drawn from the FLIP-II study, a prospective two-cohort study conducted to validate the risk factors associated for RSV infection hospitalizations in preterm infants (32^day 1^-35^day 0^ wGA) [14]. Data of the palivizumab effectiveness on preventing hospital admission comparing to non-prophylaxis in the population subgroups were collected from a further study [33].

**Table 1:** Clinical inputs parameter estimates

Parameter	Annual probabilities			
	Non-prophylaxis	Source	Palivizumab	Source
Probability of hospital admission due RSV infection				
Total population	4.10%	[14]	1.30%	[14]
Subgroup A	18.40%	[33]	9.50%	[33]
Subgroup B	10.60%		3.60%	
Subgroup C	10.20%		2.90%	
Probability of emergency visits prior to hospital admission				
	17.80%	[14]	17.80%	[14]
Probability of death related to hospital admission				
	2.33%	[3]	2.33%	[3]
Probability of additional hospitalizations due to new RSV infection				
	2.47%	[14]	2.47%	[14]
Probability of recurrent wheezing in hospitalized patients.				
Year 2	41.43%	[15]	18.43%	[15]
Year 3	29.27%		11.05%	
Year 4	18.55%		6.12%	
Year 5	15.00%		4.39%	
Year 6	12.39%		3.25%	
Probability of recurrent wheeze in no-hospitalized patients				
Year 2	12.09%	[15]	5.38%	[15]
Year 3	15.36%		5.80%	
Year 4	12.57%		4.15%	
Year 5	9.31%		2.73%	
Year 6	9.66%		2.53%	

Subgroup A includes preterm infants with 2 major risk factors and 2 minor risk factors; subgroup B, 2 major risk factors and 1 minor risk factors; subgroup C, 2 major risk factors [33]

Major factors: chronological age less than 10 weeks at the beginning of RSV season or being born during the first 10 weeks of the season; school-age siblings or day-care attendance. Minor factors: mother smoking during pregnancy and male gender

Additional hospitalizations rate due to new RSV infection (2.47%) and ICU admission rate (17.80%) were also extracted from FLIP-II study whereas in-hospital mortality rate (2.33%) were obtained from a retrospective study conducted in Spain [3]. Since no information regarding the effect of palivizumab were found on this parameters in Spanish observational studies for the particular population of preterm infants 32^day 1^ – 35^day 0^ wGA, the same parameter estimates for both alternatives were used. The model assumed that all patients experienced paediatric emergency visit prior hospital admission. A post-hoc analysis of FLIP-II study provided an average length of stay (LOS) of 6 days, which was further used to determine hospital admission costs. LOS of additional hospitalizations due to new RSV infection was assumed to be the same than for hospital admissions.

Deaths were associated to ICU admission. Due to clinical considerations, we considered that hospital admission and additional hospitalizations due to new RSV infection occurred during year 1. Consequently, it was also assumed that mortality occurred in the period 0–3 months for hospital admission and in the period 6–9 months for additional hospitalization due to new RSV infection.

The probabilities of recurrent wheezing either in previously hospitalized and non-hospitalized children (from 2 to 6 years of age) were retrieved from an observational study conducted in Spain: the SPRING study [15]**.** However, the information about the effect of palivizumab is very limited in the SPRING study, so data at 12, 24 and 36 months from the clinical evidence [17–19] were fitted to an algorithmic distribution function to get the palivizumab effect on recurrent wheezing over the 6 years period (see Additional file 1).

### Cost estimation

Total costs estimation included the pharmaceutical and administration costs, hospital admissions, and recurrent wheezing management costs. To estimate the resource consumption, either only direct costs (NHS perspective) or both direct and indirect (societal perspective) were considered.

The mean acquisition cost was calculated on the basis of published ex-factory prices (EFP) [34] for palivizumab (Synagis®) adjusted with the 15% mandatory deduction [35] applicable in November 2016.

The recommended dose of palivizumab is 15 mg/kg of body weight, given once a month during anticipated periods of RSV risk in the community [16]**.** Average dose administered were then estimated by assuming an average weight of 4.356 kg and an average number of injections of 3.88 per RSV season as reported in FLIP-II [28]**.** Since palivizumab is administered by parenteral via, it was assumed that a nurse consultation was required for each drug administration.

The health resources unitary costs were obtained from a Spanish national health costs database [36]. All costs are expressed in Euros and referred to 2016 year values. No robust evidence on recurrent wheezing costs in preterm infants RSV- infected were found in our bibliographic search, so direct and indirect costs for the management of asthma in paediatric patients were adopted [37] (Table 2).

**Table 2:** Unitary costs (€, 2016) and parameters used in the model

Parameter	Resource consumption	Source
Prophylaxis costs		
Pharmaceutical cost		
Palivizumab (Synagis ®) 50 mg per vial (€)	511.66	[34]
Palivizumab (Synagis ®) 100 mg per vial (€)	849.64	[34]
Average pharmaceutical cost (EFP/mg) (€)	7.30	
Average dose (mg/preterm infant) per injection	65.34	
Administration cost		
Average number of injections	3.88	[28]
Administration cost per injection (€)-	9.58	[36]
Hospitalization cost		
Daily cost in paediatric ward (€)	641.06	[36]
Hospital length of stay (days)	6	Post-hoc analysis FLIP II study [14]
ICU related costs		
Daily costs in ICU paediatric ward (€)	2,286.28	[36]
ICU length of stay (days) cost per visit	5	[14]
Emergency visit prior hospitalization cost		
Daily cost of emergency ward (€)	103.95	[36]
Annual recurrent wheezing management costs		
Direct cost (€)	749.57	[37]
Indirect cost (€)	498.62	[37]

*EFP:* ex-factory price; *ICU:* intensive care unit

### Utilities

The utility values applied to LYG for QALY calculation were collected from the literature. Baseline utility value was 0.95 whereas a 0.88 utility value was used for hospitalization [38]. During the literature searching none publication related to utilities for recurrent wheezing RSV infection-related was identified, so equivalence of health related quality of life between both pathologies was assumed. Then, the utility values reported in children by *Chiou* et al. from mild (0.79) for and moderate asthma symptoms were assigned to recurrent wheezing up to year 4 and year 5–6 [39]**.**

### Sensitivity analyses

To assess robustness of the model an one-way sensitivity analyses (SA) was performed to the base case parameters with the greatest level of uncertainty: average number of injections (5 doses administration); emergency visit rate prior hospital admission, hospital related costs and direct recurrent wheezing costs (variations of +/− 50%). For those clinical inputs where no local evidence on palivizumab effectiveness were found, parameter estimates from international studies were used and tested in the one-way SA: ICU admission rate (30.00% non-prophylaxis vs 11.10% palivizumab group) [26]; in-hospital mortality (0.13% non-prophylaxis vs 0.09% palivizumab group) [40]**.** Alternative scenarios modifying the discount rate were also tested (3.0% for costs and 1.5% benefits [41]; 5% for both costs and benefits; no discount rate).

A probabilistic sensitivity analysis (PSA) was also performed by using 1,000 Montecarlo simulations. Beta distribution were applied for clinical inputs and utility values; gamma distributions for costs.

## Results

### Base case

At the end of year 6, palivizumab provided higher health benefits than the non-prophylaxis strategy (5.26 vs 5.19 QALYs respectively), yielding a difference of 0.0731 QALYs. Palivizumab total costs represented €2,110.71 comparing to €671.68 in the non-prophylaxis group. Prophylaxis cost was only associated to palivizumab group (€1,886.78). Hospital admission, emergency visit costs and recurrent wheezing annual management costs were lower in the palivizumab comparing to non-prophylaxis group (Table 3). The resulting ICUR was €19,697.69/QALY gained, which means that palivizumab could be considered a cost-effective strategy assuming the common willingness-to pay threshold in Spain (€30,000/QALY gained) [42]. It could be also considered cost-effective with an even more restrictive threshold of €25,000/QALY gained recently proposed by a Health Technology Assessment Network [43]. The ICUR resulting from the population subgroups analysis according to the risk factors associated (major and minors) were €11,550.37; €14,177.18 and €13,937.61 per QALY gained for subgroups A, B and C respectively (Table 4).

**Table 3:** Base case results

	Non-prophylaxis	Palivizumab	Incremental palivizumab vs non-prophylaxis.
QALYs	5.19	5.26	0.0731
COSTS			
Total costs	€671.68	€2,110.71	€1,439.03
Prophylaxis costs (pharmaceutical and administration)	€0.00	€1,886.78	€1,886.78
Hospital related costs	€246.44	€78.14	€-168.30
Recurrent wheezing management costs	€425.23	€145.78	€-279.45
ICUR (€/QALY gained with palivizumab vs non-prophylaxis	1,9697.69		

*ICUR:* incremental cost utility ratio; *QALY:* quality adjusted life years

**Table 4:** Results of the population subgroups according to the risk factors associated

	Population subgroup A			Population subgroup B			Population subgroup C		
	Non-prophylaxis	Palivizumab	Incremental palivizumab vs non-prophylaxis.	Non-prophylaxis	Palivizumab	Incremental palivizumab vs non-prophylaxis.	Non-prophylaxis	Palivizumab	Incremental palivizumab vs non-prophylaxis.
Effectiveness									
QALYs	5.16	5.25	0.0889	5.18	5.26	0.0821	5.18	5.26	0.0822
Costs									
Total costs	€1,589.57	€2,616.92	€1,027.34	€1,088.90	€2,252.69	€1,163.79	€1,063.23	€2,209.48	€1,146.25
Prophylaxis costs	€0.00	€1,886.78	€1,886.78	€0.00 €	€1,886.78	€1,886.78	€0.00	€1,886.78	€1,886.78
Hospital related costs^a^	€1,105.98 €	€571.02	€-534.96	€637.14	€216.39	€-420.75	€613.10	€174.31	€-438.79
Recurrent wheezing costs	€483.59	€159.11	€-324.48	€451.76	€149.52 €	€-302.24	€450.13	€148.38	€-301.74
ICUR (€/QALY gained)	11,550.37			14,177.18			13,937.61		

QALY: Quality adjusted life years; Subgroup A: 2 major risk factors and 2 minor risk factors; Subgroup B: 2 major factors and 1 minor factor; Subgroup C: 2 major factors; Major factors included chronological age less than10 weeks at the beginning of RSV season or being born during the first 10 weeks of the season; school-age siblings or day-care attendance whereas minor factors included mother smoking during pregnancy and male gender [33]. Major factors: chronological age less than 10 weeks at the beginning of RSV season or being born during the first 10 weeks of the season; school-age siblings or day-care attendance. Minor factors: mother smoking during pregnancy and male gender

^a^Hospital related costs included emergency visit prior hospitalization cost, hospital and ICU admission costs

From the societal perspective, the difference in total cost between the palivizumab-based strategy and the non-prophylaxis were lesser comparing with NHS perspective (€1,253.14), which resulted in a decrease of the ICUR (ICUR = €17,153.16/QALY gained).

### Sensitivity analysis

**Fig. 2:**
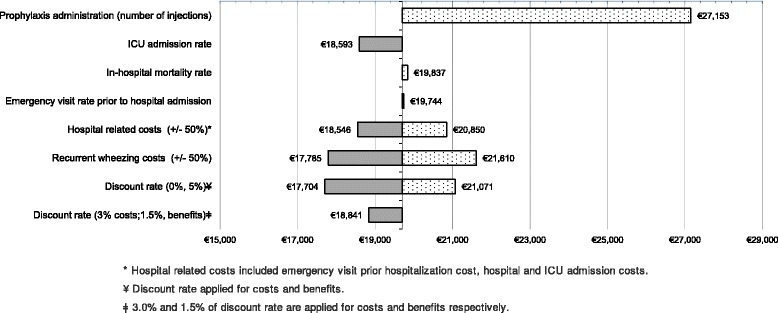
Tornado diagram of the one-way SA

Regarding the one-way SA, palivizumab remained as a cost-effective option in all the scenarios tested. The highest ICUR were found when the average number of injections increased to 5 (€27,135/QALY gained). The variations in the in-hospital mortality rates (€19,837/QALY gained) and the emergency visits prior to hospital admission (€19,744/QALY gained) had a low impact on the increase of ICUR. The scenarios that tested an increase of 50% costs (recurrent wheezing and hospital related-costs) resulted in a drop of ICUR values whereas when we applied a decrease of 50% in those scenarios it results in an augmentation of the base-case ICUR. The ICU admission rates variations and discount rate of 3.0% costs and 1.5% for benefits decreased slightly with respect ICUR base-case (Table 5, Fig. 2).

**Table 5:** One-way SA results

Parameters	Base case values		One-way SA Values		Incremental costs	Incremental QALY	ICUR (€/QALY gained)
	Non prophylaxis	Palivizumab	Non- prophylaxis	Palivizumab			
Number of palivizumab injections per RSV season	–	3.88	–	5	€1,983.67	0.0731	27,152.79
Risk of ICU admission	17.80%	17.80%	30.00%	11.10%	€1,370.71	0.0737	18,592.52
In-hospital mortality	2.33%	2.33%	0.13%	0.09%	€1,438.90	0.0725	19,836.81
Proportion of patients that experience emergency visit prior to hospital admission	100.00%		50.00%		€1,440.49	0.0731	19,717.70
Hospital related costs* (+/−50%)	€3,031.30		+50.00% (€4.546,95)		€1,354.88	0.0731	18,545.80
			−50.00% (€1515.65)		€1,523.18	0.0731	20,849.55
Direct recurrent wheezing management costs (+/−50%)	€749.57		+50.00% (€1124.36)		€1,299.30	0.0731	17,785.09
			−50.00% (€.374.79)		€1,578.76	0.0731	21,610.25
Annual discount rate	Costs	Benefits	Costs	Benefits			
	3.0%		0.0%		€1,415.22	0.0799	17,704.00
			5.0%		€1,453.22	0.0690	21,071.19
			3.0%	1.50%	€1,439.03	0.0764	18,841.25

*ICU*: intensive care unit; *ICUR*: incremental cost-utility ratio; *QALY* quality adjusted life years, *RSV* respiratory syncytial virus, *SA* sensitivity analysis

*Hospital related costs included emergency visit prior hospitalization cost, hospital and ICU admission costs

**Fig. 3:**
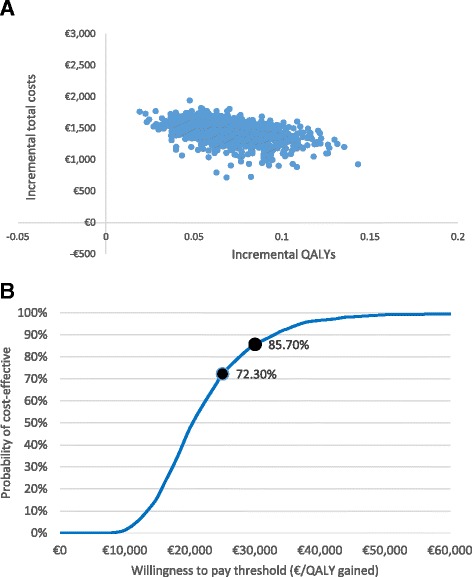
Probabilistic sensitivity analysis. **a** Cost-effectiveness plane. **b** Acceptability curve

Finally, a cost effectiveness plane (Fig. 3a) and cost-effectiveness acceptability curve (Fig. 3b) were used to show PSA results. Out of 1000 Montecarlo simulations, 85.70% of the cases presented an ICUR under a €30,000/QALY gained threshold [42], whereas in 72.30% of the cases they were under a €25,000/QALY gained value [43].

## Discussion

This study used an analytic model to assess the efficiency of a palivizumab taking into account the most recent clinical evidence on RSV infections in preterm infants (32^day 1^-35^day 0^ wGA) in Spain.

The analysis showed that palivizumab could be considered a cost-effective strategy to prevent the RSV infections and its sequelae independently of the perspective used. The ICUR obtained from the NHS perspective (€19,697.69/QALY gained) was lower than the commonly acceptable threshold of €30,000/QALY gained considered in Spain [42] and the more restrictive threshold recently published of €25,000/QALY gained [43]. When recurrent wheezing indirect costs were considered in the total population it resulted in an even more favourable ICUR (€17,153.16/QALY gained). Moreover, this work also examined the efficiency of palivizumab in 3 population subgroups according to the risk factors validated in FLIP-II study, whose results were also consistent with the base-case analysis.

The model is sensitive to an increase of number of injections when compared with the base-case analysis, since the ICUR obtained when we used 5 average doses of palivizumab leads to a considerably less favourable ICUR (€27,152.79 vs €19,697.69)/QALY gained). In contrast, if the discount rate for costs and benefits is not applied (€17,704.00/QALY gained) or recurrent wheezing cost is increased by 50% we found the most favourable ICUR (€17,785.09/QALY gained).

Previous economic evaluations underwent in Spain presented heterogeneous results [28–30], but only *Lazaro de Mercado* et al. [28] study were addressed to the particular population analysed in this work (32^day 1^-35^day 0^ wGA). It resulted in €13,849 and €4605/QALY gained(€ 2006) from the NHS and societal perspective respectively, a substantially lower ICUR compared to the one obtained in the present analysis. However, the data used and the assumptions made in the analytic model developed by *Lazaro* et al. [28] can explain this differences. First off, it did not include country specific data on hospital admission and mortality rates, which were drawn from the IMPACT-RSV study [44] and Canadian database for deaths [45]. Furthermore, it did not consider recurrent wheezing as a clinical parameter that can affect to hospitalization but just as a factor to convert LGY to QALY. In contrast, the parameters estimates in the present study not only are based on the Spanish specific data (i.e.: hospitalization admission, ICU and mortality rates), but also it used empirical data for estimates on long term recurrent wheezing rates in preterm from the SPRING study and subsequently measure the impact of recurrent wheezing on resource consumption and health outcomes.

Comparisons with studies at international level should be precautionary done, based on potential differences on methodology (time horizon, discount rate, population) and drug prices. The results of the present analysis are in line with other studies which assessed palivizumab in similar populations, identifying palivizumab as a cost-effective strategy in Austria [20] (ICUR = €21,864/QALY gained in 33-35wGA population, 2010 year values), Netherlands [21] (ICUR = €20,236/QALY gained in 32–35 wGA population with bronchopulmonary dysplasia and €7067/QALY gained in 32–35 wGA population with chronic heart disease, 2006 year values), UK [23] (ICUR = €16,720/QALY gained in 32–35 wGA population with bronchopulmonary dysplasia and €6664/QALY gained in 32–35 wGA population with chronic heart disease, 2003 year values), and USA (ICUR = $79,479/QALY gained [25] and ICUR = $38,244/QALY gained [26] in 32–35 wGA population with American Academy of Pediatrics-AAP- 2006 criteria, 2010 year values). In other study performed in UK [24], ICUR resulted £99,056/QALY gained (2010 year values) in 33–35 wGA infants and based on a threshold of £30,000/QALY gained was defined as a not-cost-effective option.

Some assumptions that may limit the strength of the model were applied. First, due to the lack of evidence in preterm infants RSV- infected, we used utility values and costs reported in the management of asthma in paediatric patients. For the same reason, we extrapolated the existing evidence on palivizumab effect at 12, 24 and 36 months and further applied the relative risk from SPRING study to obtain the recurrent wheezing rates in hospitalized and non-hospitalized RSV- infected infants over a 6 years period (see additional file 1).

Secondly, due to the lack of information of palivizumab effectiveness for the particular population of preterm infants 32^day 1^ – 35^day 0^ wGA in local observational studies, we assumed no effect of palivizumab on ICU admission, mortality rates and LOS.

In spite of the fact that assuming the same rates for palivizumab and non-prophylaxis group has been reported as a conservative approach in previous economic evaluations [23, 25, 26], we run an one-way SA to assess the impact of using estimates for palivizumab and non-prophylaxis from international studies [26, 40] on these clinical inputs. The ICU admission estimates reported *by Weiner* et al. (30.00% non-prophylaxis vs 11.10% palivizumab; ICUR = €18,592.52/QALY gained) [26] and in-hospital mortality rates from *Checchia* et al. (0.13% non-prophylaxis vs 0.09% palivizumab; ICUR = €€19,836.81/QALY gained) [40] showed no significant impact on the resulting ICUR comparing to the base case (€19,697.69/QALY gained).

Length of hospital stay could be a variable parameter, related to differences in clinical practice. For the base case an average of 6 days at paediatric ward or 5 days at ICU was considered given a hospital-related cost. This cost was varied +/− 50% in the SA capturing the potential effect of shorter or longer admissions. Even for the best clinical scenario with the shortest hospitalization (decrease of 50% of hospital cost) the ICUR kept under the acceptable threshold.

Besides the results for subgroups of the present analysis reside on a risk stratification derived from a Spanish epidemiological study, that it is not elsewhere spread, but could be understood as illustrative for identification of existing subpopulations associated to better or worse outcomes.

Usually, pharmaceutical cost are among the main drivers on cost-effectiveness analysis. In Spain, maximum prices for reimbursed drugs are fixed at national level, but some local agreements for price reductions could be established at regional or hospital level.The ICURs resulting from the model here described, derived from the official maximum reimbursed price. Lower ICUR values would had been expected if lower drug prices (related to the mentioned agreements) applied.

Despite of all of the mentioned limitations, the variations made for the rest of parameters tested in the one-way SA and the fact that almost 90% of the 1000 simulations run in the PSA remained below the threshold of €30,000/QALY confirmed the robustness of the model.

In economic evaluation of health technologies a specific threshold of willingness to pay is required for concluding whether the assessed strategy is cost-effective versus the alternative one or not. Facing the absence to date of an official value stated in Spain, for the present analysis a threshold of €30,000 per additional QALY was used as main reference, coincident with the value used in the vast (66%) of the economic evaluations performed for Spain [46]. Mention to an alternative threshold, recently published [43] is also performed.

To the best knowledge of the authors, this work represents the first cost-effectiveness study including evidence of the long-term effects of palivizumab in preterm infants 32^day 1^ – 35^day 0^ wGA RSV- infected in Spain. In this sense, the American Academy of Paediatrics launched in 2014 a document by which prophylaxis with palivizumab is not recommended either to preterm infants to reduce subsequent episodes of wheezing or for otherwise healthy infants born at or after 29^day 0^ wGA. [47] However, no evidence were given to support those modifications [48] including the recent evidence of the long term effect of palivizumab on recurrent wheezing in preterm infants 32^day 1^-35^day 0^ RSV-infected, which were never evaluated in cost-effectiveness studies [49]. Therefore the favourable ICUR resulting from our analysis may contribute to support the position paper of the Standards Committee of the Spanish Neonatology Society (SENeo).

## Conclusion

Recent evidence on the long-term effect of recurrent wheezing in Spain allowed to provide an updated economic evaluation of the prophylaxis with palivizumab in preterm infants.

In the light favourable ICUR obtained, palivizumab is efficient for preventing from RSV infections in preterm infants 32^day 1^-35^day 0^ wGA in Spain, including specific high risk subgroups.

## Additional file

Additional file 1: Figure S1. Curve fitting to get the palivizumab effect on recurrent wheezing over 61 years by using RR values from the literature. (DOCX 19 kb)

## Abbreviations

EFP: Ex-factory price
ICU: Intensive care unit
ICUR: Incremental cost utility ratio
LOS: Length of stay
NHS: National Health System
PSA: Probabilistic sensitivity analysis
QALY: Quality Adjusted Life Years
RSV: Respiratory Syncytial Virus
SA: Sensitivity analysis
SENeo: Spanish Neonatology Society
wGA: Weeks of gestational age
