# Supplementary material for: Cost-utility analysis of Palivizumab for Respiratory Syncytial Virus infection prophylaxis in preterm infants: update based on the clinical evidence in Spain
Source: BMC Infect Dis. 2017 Oct 17;17:687. doi: 10.1186/s12879-017-2803-0 (PMC5645982; doi:10.1186/s12879-017-2803-0)
Supplement: Additional file 1: Figure S1. — Curve fitting to get the palivizumab effect on recurrent wheezing over 61 years by using RR values from the literature. (DOCX 19 kb) [file 12879_2017_2803_MOESM1_ESM.docx]

# Additional file 1

**Figure S1 Curve fitting to get the palivizumab effect on recurrent wheezing over 6 years by using RR values from the literature.**

To obtain the efficacy rates of palivizumab in the prevention of recurrent wheezing, we performed a 2-step analysis. First, different distributions functions (polynomic, exponential, logarithmic and potential) were generated based on the risk reduction associated to palivizumab versus no prophylaxis at 12 **[1]**, 24 **[2]**, 36 months **[3]** (Figure 4). Secondly, once was shown the algorithmic function best fitted the generated functions and offered the more reliable data, the relative risk reduction was applied to the data on recurrent wheezing in preterm infants 32 ^day1^-35 ^day0^ in the Spanish population from the SPRING study **[4]**.

# Abbreviations

RSV: Respiratory Syncytial Virus

# References

1. Blanken MO, Rovers MM, Molenaar JM, Winkler-Seinstra PL, Meijer A, Kimpen JL, et al. Respiratory syncytial virus and recurrent wheeze in healthy preterm infants. N Engl J Med. 2013;368:1791-9.
2. Simoes EA, Groothuis JR, Carbonell-Estrany X, Rieger CH, Mitchell I, Fredrick LM, et al. Palivizumab prophylaxis, respiratory syncytial virus, and subsequent recurrent wheezing. J Pediatr. 2007;151(1):34-42.
3. Yoshihara S, Kusuda S, Mochizuki H, Okada K, Nishima S, Simões EA, et al. Effect of palivizumab prophylaxis on subsequent recurrent wheezing in preterm infants. Pediatrics. 2013;132:811-8
4. Carbonell-Estrany X, Pérez-Yarza EG, García LS, Guzmán Cabañas JM, Bòria EV, Atienza BB; IRIS (Infección Respiratoria Infantil por Virus Respiratorio Sincitial) Study Group. Long-Term Burden and Respiratory Effects of Respiratory Syncytial Virus Hospitalization in Preterm Infants-The SPRING Study. PLoS One. 2015;8;10(5).
